# Optical control of the hypothalamic arcuate nucleus kisspeptin neuronal network with a photoswitchable peptide that drives luteinizing hormone release in female mice

**DOI:** 10.1111/jne.70255

**Published:** 2026-09-08

**Authors:** Jian Qiu, Yanyan Lin, Emily Bartram, Janelle M. Tobias, Antonio Muñoz, Xiao Feng Li, Victoria S. Halls, James A. Frank, Martin J. Kelly, Kevin T. O'Byrne

**Affiliations:** ^1^ Department of Chemical Physiology and Biochemistry Oregon Health & Science University Portland Oregon USA; ^2^ Department of Women and Children's Health, School of Life Course and Population Sciences King's College London, Guy's Campus London UK; ^3^ Oregon Health & Science University Vollum Institute Portland Oregon USA; ^4^ Medicinal Chemistry Core Facility Oregon Health & Science University Portland Oregon USA

**Keywords:** *azo*‐senktide, kisspeptin, LH, peptide, photoswitchable

## Abstract

Pulsatile gonadotropin‐releasing hormone (GnRH) and luteinizing hormone (LH) secretion are essential for reproductive function and are thought to be governed by arcuate nucleus kisspeptin Kiss1^ARH^ neurons via neurokinin B (NKB) signaling through TacR3. However, discrepancies between ex vivo and in vivo pharmacology limit mechanistic understanding of how TacR3 activation regulates pulsatile hormone release. We developed and synthesized a photoswitchable TacR3 agonist (*azo*‐senktide) and characterized its photochemical and pharmacological properties using UV–Vis spectroscopy, TacR3‐expressing HEK293T cells, electrophysiology in mouse hypothalamic slices, and in vivo optofluidic delivery combined with light stimulation to assess LH secretion in freely moving mice. *Azo*‐senktide reversibly photoisomerized between inactive (trans) and more potent (cis) conformations. In cells, light activation enhanced TacR3‐mediated Ca^2+^ signaling. In brain slices, photostimulation depolarized and increased firing of Kiss1^ARH^ neurons through a mechanism consistent with the established TacR3–TRPC5 signaling pathway. In vivo, optical activation of *azo*‐senktide in the arcuate nucleus elicited a time‐locked LH pulse at an intermediate dose, whereas lower or higher doses were ineffective, indicating a nonlinear, dose‐dependent response. These findings demonstrate that temporally gated TacR3 activation via photoswitchable *azo*‐senktide evokes an increase in LH, revealing that neurokinin B signaling in the Kiss1^ARH^ network depends on activation dynamics, reconciles inconsistencies, and establishes photopharmacology as a powerful approach for probing neuroendocrine timing mechanisms.

## 1 INTRODUCTION

Pulsatile neuronal activity and hormone release are a key characteristic of neuroendocrine signaling. As opposed to continuous, tonic secretion, hypothalamic neural circuits release neuropeptides in rhythmic bursts. This preserves receptor sensitivity in target tissues, allows frequency‐dependent modulation of downstream effector pathways, and allows a single neuropeptide signal to encode diverse physiological information. Pulsatile signaling is a hallmark of the hypothalamic–pituitary–gonadal (HPG) axis. Rhythmic release of gonadotropin‐releasing hormone (GnRH) into the median eminence drives pulsatile release of luteinizing hormone (LH) from the pituitary gland, a pattern that is essential for gametogenesis and reproduction. Despite its physiological importance, the upstream hypothalamic neural circuits that coordinate rhythmic GnRH and LH secretion remain poorly defined. Recently, the arcuate nucleus kisspeptin (Kiss1^ARH^) neurons have emerged as leading candidates as the pulse generator neurons that dictate pulsatile GnRH secretion.^1^, ^2^ Optogenetic studies provided the first evidence of their pulse‐generator function, where photostimulation of channelrhodopsin (ChR2)‐expressing Kiss1^ARH^ neurons led to pulsatile LH release in mice.^3^ Electrophysiological studies demonstrated that this activity was dependent on the release of two co‐expressed neuropeptides: neurokinin B (NKB), which activates the tachykinin receptor 3 (TacR3) and has been shown to depolarize postsynaptic Kiss1^ARH^ neurons through activation of TRPC5 channels^4^, ^5^, ^6^; and dynorphin, which activates presynaptic kappa‐opioid receptors to limit the release of NKB to discrete bursts of activity.^4^ These two peptide neurotransmitters coordinate the synchronous firing of Kiss1^ARH^ neurons that governs the pulsatile release of GnRH into the median eminence.^4^, ^7^, ^8^

Despite these technological advances that have enabled our increased understanding of pulse‐generator circuits, key questions remain due to a pharmacological paradox that emerged when targeting these pathways with selective neuropeptide receptor agonists. In ex vivo slice preparations, application of a selective TacR3 agonist senktide robustly depolarizes Kiss1^ARH^ neurons and drives synchronization and hormone release through the downstream activation of TRPC channels.^4^, ^5^, ^6^ In vivo, however, delivery of senktide has been shown to either increase^9^ or decrease^10^, ^11^ LH release, depending on the dose and injection location, although the inhibitory response while observed in rats has not been confirmed in mice, which could potentially represent a species difference. Nevertheless, this disagreement between the ex vivo and in vivo data presents a barrier to understanding how neuropeptides dictate pulse‐generator activity. Optogenetics uses exogenous signaling proteins to depolarize cells, which does not inform on endogenous signaling pathways; and standard pharmacological manipulation lacks the spatiotemporal precision that is needed to mimic pulsatile neuropeptide release and distinguish the effects of receptor activation from desensitization and feedback pathways.

Photopharmacology, which involves the addition of photoswitchable functional groups onto bioactive signaling molecules, offers a powerful strategy to overcome these limitations. In this approach, a photoswitchable motif (typically an azobenzene) is incorporated into a bioactive ligand that allows the conformation of the ligand, and consequently its binding affinity at endogenously‐expressed receptors, to be reversibly controlled by light.^12^ A variety of photoswitchable peptides have been described^13^, ^14^ and are useful tools to reversibly control biological functions. However, few probes currently exist for manipulating neuropeptide signaling in intact neural circuits. In this study, we present the design, synthesis, and validation of a photoswitchable TacR3 agonist based on the senktide scaffold, coined *azo*‐senktide, that contains an azobenzene photoswitch attached via a phenylalanine sidechain. We characterize *azo*‐senktide's light‐dependent pharmacology in cells heterologously expressing TacR3, confirm its activity on Kiss1^ARH^ neurons in ex vivo brain slices, and demonstrate the optical control of LH pulse release in vivo following acute delivery and photostimulation in the arcuate nucleus. This novel probe sheds light on the longstanding disagreement between the ex vivo and in vivo studies, revealing how the dynamics of TacR3 activation determines whether NKB signaling is able to excite the pulse‐generator circuit.

## 2 MATERIALS AND METHODS

### 2.1 Chemicals and reagents

All standard solvents, salts, and acids were purchased from Fisher Scientific. Standard Fmoc‐protected amino acids were purchased from Novabiochem (Millipore Sigma). Fmoc‐Phe(4‐N=NPh)—OH was purchased from Watanabe Chemical Industries, Ltd. Fmoc Rink Amide ProTide low‐loading (LL) resin and Oxyma were purchased from CEM Corporation. Triisopropyl silane and *N,N′*‐diisopropylcarbodiimide (DIC) were purchased from TCI America. Piperidine was purchased from Sigma‐Aldrich. For peptide synthesis, all solutions were prepared in *N,N′‐*dimethylformamide.

#### Synthesis and characterization of azo‐senktid

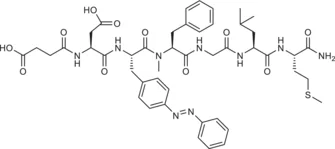

*Azo*‐Senktide was synthesized at a 0.1 mmol scale using a CEM Liberty Blue Automated Microwave Peptide Synthesizer. For all amino acids, the Liberty Blue standard 0.1 mmol coupling method was used (2.5 mL 0.2 M amino acid, 1 mL 1 M DIC, 0.5 mL 1 M Oxyma, 90°C, 65 min) and deprotection was carried out using Liberty Blue standard deprotection (20% piperidine/DMF, 90°C, 85 min).

After synthesis, the resin was placed in a fritted syringe and the peptide was cleaved by adding a mixture of trifluoroacetic acid, triisopropyl silane, and water (96:2:2, 6 mL). This mixture was shaken for 30 min at 200 rpm and 42°C in a New Brunswick Scientific C25KC Incubator Shaker and the solution was then eluted into a 20‐mL scintillation vial. The resin was washed with trifluoroacetic acid (4 mL) and the combined eluents were reduced to dryness under reduced pressure. The residue was dissolved in a mixture of acetonitrile, water, and formic acid (49.95:49.95:0.1, 4 mL) and lyophilized for 18 h.

The residue was dissolved in a mixture of acetonitrile, water, and formic acid (49.95:49.95:0.1, 15 mL) and purified via high‐performance liquid chromatography (HPLC) on an Agilent 1260 infinity with Agilent Pursuit XRs Column (100 Å C18, 30 × 250 mm, 5 μm) in three runs injecting 5 mL each time. The HPLC run was carried out in a 25 min linear gradient from 50% to 100% B at a flow rate of 45 mL/min using water (A) and acetonitrile (B) each with 0.1% formic acid as mobile phase. Product fractions were identified using an LTQ Velos MS, pooled and lyophilized. *Azo*‐Senktide: Yield 35 mg (40%). ESI+ calculated m/z: [M + H]^+^ = 946.75, found: [M + H]^+^ = 946.08/946.10 (*cis*/*trans*).

### 2.2 UV–Vis spectroscopy

*Azo*‐senktide was dissolved at 40 μM in dimethylsulfoxide (DMSO) and placed in a Quartz cuvette (1 mL, 10 mm light path). A deuterium‐halogen light source (Ocean Optics #DH‐2000) was attached to an optical fiber (400 μm diameter) and transmitted light through the sample. Spectra were recorded using a Flame UV–Vis–ES spectrophotometer (FLMT05021, Ocean Insight) using OceanView (Version 2.0.7) Software. Photoswitching was initiated by irradiation with 365 nm (Thorlabs M365FP1) and 470 nm (Thorlabs M470F4) fiber‐coupled light‐emitting diodes (LEDs) guided through a fiber‐optic cable (Thorlabs #FP400URT, 400 μm diameter, 0.50 numerical aperture) and optical cannula (400 μm, Thorlabs). The cannula was pointed directly into the top of the sample for illumination. A detailed depiction of the experimental setup for UV–Vis experiments, alongside the emission spectra for our 365‐ and 470‐nm LEDs, is provided in Garza et al.^15^

### 2.3 Cell culture

HEK293T cells were grown in D10 media (high‐glucose Dulbecco's Modified Eagle Medium (DMEM) [Gibco, #11965‐092], 10 vol% fetal bovine serum (FBS) [HyClone's USDA tested, #SH30910], 0.5 vol% Penicillin‐strep [Gibco, #15070]) at 37°C and 5% CO_2_. For imaging experiments, HEK293T cells were plated at a density of 40,000 cells per well on 8‐well glass bottom chambered coverslips (Ibidi, #0827‐90). Twenty‐four hours later, the cells were starved in Opti‐MEM™ (250 μL) for 3–4 h before adding the transfection mixture containing (per well): 30 μL Opti‐MEM™, 0.5 μL Lipofectamine‐2000 (Fisher Scientific, #11668019) and cDNA (150 ng R‐GECO and 150 ng hTacR3). The cDNA clone for human TacR3 was obtained from the cDNA Resource Center (www.cdna.org, #TACR300000). The cells were then incubated at 37°C and 5% CO_2_ for 18–24 h before exchanging the transfection mixture with D10 media. Microscopy experiments were performed 18‐ to 48‐h post‐transfection.

### 2.4 Confocal microscopy

Live‐cell confocal imaging was achieved using an Olympus Fluoview 1200 laser scanning confocal microscope equipped with an environmental box keeping the cells at 37°C and 5% CO_2_. Time‐lapse fluorescence images were acquired with a 20×/0.75 objective (Olympus UPlanSApo) at 512 × 512‐pixel resolution and scan rate of 4 s/frame. R‐GECO was excited with a 559‐nm laser (1–3% laser power) and the emission was collected at 570–670 nm. Compound solutions (stocks dissolved at 1000× in DMSO) were diluted to 1× with imaging buffer (contains in mM: 185 NaCl, 1.2 CaCl_2_, 1.2 MgCl_2_, 1.2 K_2_HPO_4_, 20 4‐(2‐hydroxyethyl)‐1‐piperazineethanesulfonic acid (HEPES), adjusted to pH 7.4 with NaOH). d‐glucose was supplemented at 20 mM and pipetted directly into the imaging well. Vehicle controls were performed using an identical procedure, adding a final concentration of DMSO (0.1 vol%) with no compound. Photoswitching was achieved using a 375‐nm laser (PicoQuant, PDL 800‐D, ~95 μW output from the objective) triggered using the quench function in the Olympus software. Ionomycin (10 μM) was added at the end of each experiment to maximize the R‐GECO fluorescence intensity. Fluorescence intensity values in each experimental video were collected and analyzed with Fiji,^16^ and the data processed in Excel and MATLAB using in‐house written scripts (available on request). Oval‐shaped regions were drawn over cells to measure the fluorescence intensity. The number of cells (*N*) and the number of individual videos (*T*, biological replicates) represented in each data panel is reported in the figure caption.

### 2.5 Animals

All the animal procedures described in this study were performed in accordance with institutional guidelines based on National Institutes of Health standards and approved by the Institutional Animal Care and Use Committee at Oregon Health and Science University (OHSU) or in accordance with the United Kingdom Home Office Animals (Scientific Procedures) Act 1986 and approved by the Animal Welfare and Ethical Review Body Committee at King's College London.

### 2.6 Mice for in vitro electrophysiology experiments

*Kiss1* ^*Cre*^ transgenic female mice version 2^17^ were selectively bred at OHSU and KCL. All animals were maintained under controlled temperature and photoperiod (lights on at 0600 h and off at 1800 h at OHSU or 0700 h and off at 1900 h at KCL) and given free access to food (Lab Diets 5L0D) and water. All females were ovariectomized 7 days prior to an experiment.

### 2.7 AAV delivery to Kiss1^Cre^ mice

Fourteen to 21 days prior to each experiment, the *Kiss1* ^*Cre*^ mice (>60 days old) received bilateral hypothalamic arcuate nucleus (ARH) injections of a Cre‐dependent adeno‐associated viral (AAV; serotype 1) vector encoding mCherry (AAV1‐Ef1α‐DIO‐mCherry) to visualize Kiss1^ARH^ neurons. Using aseptic techniques, anesthetized female mice (1.5% isoflurane/O_2_) received a medial skin incision to expose the surface of the skull. The glass pipette with a beveled tip (diameter = 45 μm) was filled with mineral oil, loaded with an aliquot of AAV using a Nanoject II (Drummond Scientific). ARH injection coordinates were anteroposterior: −1.20 mm, mediolateral: ± 0.30 mm, dorsoventral: −5.80 mm (surface of brain *z* = 0.0 mm); 500 nL of the AAV (2.0 × 10^12^ particles/mL) was injected (100 nL/min) into each position, and the pipette left in place for 10‐min post‐injection, then slowly retracted from the brain. The skin incision was closed using Vetbond (3 M) and each mouse received analgesia (Rimadyl, 4–5 mg/kg, *s.c*.).

### 2.8 Visualized whole‐cell patch recording

Electrophysiological and photo‐switching studies were made in coronal brain slices (250 μm) containing the ARH from AAV1‐EF1α‐DIO‐mCherry injected Kiss1^Cre^ mice, which were vehicle‐treated ovariectomised (OVX) females 10 weeks of age and older as previously described.^4^, ^18^ Whole‐cell patch recordings were performed in voltage‐clamp or in current‐clamp as previously described^18^ using an Olympus BX51W1 fixed stage scope outfitted with epifluorescence and IR‐DIC video microscopy. Patch pipettes (A‐M Systems; 1.5‐μm outer diameter borosilicate glass) were pulled on a Brown/Flaming puller (Sutter Instrument, model P‐97) and filled with the following solution: 128 mM potassium gluconate, 10 mM NaCl, 1 mM MgCl_2_, 11 mM ethylene glycol‐bis(β‐aminoethyl ether)‐N,N,N′,N′‐tetraacetic acid (EGTA), 10 mM HEPES, 2 mM adenosine triphosphate (ATP), and 0.25 mM guanosine 5'‐triphosphate (GTP) adjusted to pH 7.3 with KOH; 295 mOsm. Pipette resistances ranged from 3.5 to 4 MΩ. In whole‐cell configuration, access resistance was less than 30 MΩ; the access resistance was 80% compensated. The input resistance was calculated by measuring the slope of the I–V relationship curve between −70 and −90 mV. Standard whole‐cell patch recording procedures and pharmacological testing were performed as previously described.^19^, ^20^ Electrophysiological signals were digitized with a Digidata 1440A (Axon Instruments) and the data were analyzed using p‐Clamp software (Molecular Devices, Foster City, CA). The liquid junction potential was corrected for all data analysis. For photo‐switching, a pharmacological response was evoked using a LED 375‐nm light source (0.9 mW intensity) controlled by a variable 2A driver (ThorLabs, Newton, NJ) with the light path directly delivered through an Olympus 40× water‐immersion lens.

After identifying and patching Kiss1 neurons using mCherry fluorescence, the visualization light was immediately shuttered and remained off throughout the photopharmacology experiments. Following establishment of the whole‐cell configuration, the cells were perfused with *azo*‐senktide, and 375‐nm photostimulation was applied. Thus, no mCherry excitation light was present during UV illumination, preventing any unintended photoisomerization or antagonism of the *cis*‐state by longer‐wavelength light.

### 2.9 Electrophysiological solutions/drugs

A standard artificial cerebrospinal fluid (aCSF) (in mM: 124 NaCl, 26 NaHCO_3_, 10 dextrose, 10 HEPES, 5 KCl, 2.6 NaH_2_PO_4_, 2 MgSO_4_, and 1 CaCl_2_) was used.^4^, ^19^, ^21^ All drugs were purchased from Tocris Bioscience (Minneapolis, MN) unless otherwise specified: 1 mM, tetrodotoxin (TTX) (Alomone Labs, Jerusalem, Israel); 100 μM, *azo*‐senktide; 50 mM, SB222,20; 1 mM, 100 μM, senktide and 100 mM, 2‐aminoethyl diphenylborinate (2‐APB). Stocks (1000×) were prepared in DMSO (*azo*‐senktide, SB222,20; 100 mM, senktide and 2‐APB) or water (TTX) and stored at −20°C. Aliquots of the stock solutions were stored as appropriate until needed.

### 2.10 Electrophysiology experimental design and statistical analysis

For whole‐cell patch recording experiments, one cell was recorded per slice. Two to three slices were analyzed from each Kiss1^Cre^ mouse, with at least 3–5 mice contributing to each group. Comparisons between two groups were performed using an unpaired two‐tailed Student's *t*‐test. Comparisons between more than two groups were performed using one‐way analysis of variance (ANOVA), followed by the multiple range tests as specified in the appropriate figure legends. All data were analyzed using GraphPad Prism version 6. All data are presented as mean ± standard error of the mean (SEM); data points represent individual cells. Differences were considered statistically significant if the probability of error was less than 5%.

### 2.11 Mice for in vivo experiments

Adult female C57BL6J mice (8–10 weeks) used for the experiments (*n* = 7) were purchased from Charles River Laboratories International, Inc. They were individually housed at a constant temperature (22 ± 2°C) with a consistent light/dark cycle (12‐h light, 12‐h dark, with lights on at 07:00 h). Mice were provided food and water ad libitum.

### 2.12 Surgical procedures

At 8 weeks of age, vaginal smears were performed daily between 10:00 and 12:00 h on mice for 1 week prior to the surgery; only mice with normal estrous cycles were included in the study. Mice were anesthetized using ketamine (Vetalar, 100 mg/kg, intraperitoneal injection; Pfizer, Sandwich, UK) and xylazine (Rompun, 10 mg/kg, intraperitoneal injection; Bayer, Leverkusen, Germany). All surgical procedures were performed using a motorized robot stereotaxic system (Neurostar, Tubingen, Germany). The stereotaxic coordinates used to target the ARH were obtained from the mouse brain atlas of Franklin and Paxinos^22^ (1.65 mm posterior to bregma, 0.24 mm lateral, and at a depth of 6.05 mm). A dual optofluid cannula, containing an optic fiber and fluid injection cannula (Doric Lenses Inc. Quebec, Canada), was implanted into the left ARH and fixed in place using dental cement (Super‐Bond Universal Kit, Prestige Dental, UK). All procedures were conducted under aseptic conditions.

### 2.13 In vivo azo‐senktide administration, photostimulation and blood sampling

*F*ollowing a 1‐week recovery period, mice were handled daily for 3 weeks before blood sample collection to minimize handling stress during blood sampling.^23^ Vaginal smears were continued 1 week after the surgery and throughout the experimental period to evaluate cycle stage. All experiments and blood samples were collected during the estrous stage to minimize the chance of an endogenous LH pulse occurring.^24^ Each animal was exposed to different dose or light conditions in a randomized sequence (4–6 sessions per animal), with no treatment or control condition repeated within the same animal with at least one cycle between experiments. Data collection was conducted over a period of 4–6 weeks.

On the day of the experiment, the zirconia ferrule of the implanted cannula was connected to the photostimulation source via a ceramic mating sleeve to a multimode fibre optic rotary joint patch cable (Thorlabs Ltd, Ely, UK) to allow for free movement of the mice in their home cage. An injection cannula connected to extension tubing preloaded with either the drug solution or control vehicle (artificial cerebrospinal fluid; aCSF; 250 nL) was inserted into the guide cannula of the optofluid implant immediately after connection of the fiber optic cannula. The tail tip was cut and the mice were left to habituate in their cage for 60 min. To measure LH, 4 μL of blood was taken every 5 min for 1 h from an unrestrained mouse.^23^ Blood samples were stored at −80°C until later analyses. At 25 min, an infusion of *azo*‐senktide or aCSF was initiated over a 5‐min period through the pre‐loaded tubing connected to the optofluid cannula using a 5 μL syringe (SGE, Kinesis, Redland Bay, Australia). Photostimulation was initiated at 30 min for a 2 min duration with continuous light at 365 nm, 7 mW using an LED driver (ThorLabs, New Jersey, USA). The 3 doses of *azo*‐senktide (5, 50, or 500 pmol in 250 nL aCSF) or aCSF controls were used. All experiments commenced between 10:00 and 10:30 h.

### 2.14 Validation of optofluid cannulae position

After completion of experiments, mice were anaesthetized with a lethal dose of pentobarbital (Euthatal, 200 mg/kg, intraperitoneal injection; Merial, Essex, UK) and transcardial perfusion was performed with heparinized saline for 5 min followed by ice‐cold 4% paraformaldehyde in phosphate buffer (pH 7.4) for 15 min using a pump (Minipuls, Gilson, Villiers Le Bel, France). The brains were collected and postfixed in 15% sucrose in 4% paraformaldehyde at 4°C and left to sink. Once sunk, they were transferred to 30% sucrose in phosphate‐buffered saline and left to sink. Brains were snap‐frozen in isopropanol on dry ice and stored in −80°C until further processing. Every third coronal brain section (30 μm/section) throughout the ARH region (−1.34 to −2.70 mm from bregma) was collected using a cryostat (Bright Instrument Co., Luton, UK). Sections were mounted on microscope slides, air dried, and stained with cresyl violet. Cannula placement was verified with Axioskop 2 Plus microscope equipped with Axiovision, version 4.7 (Zeiss) by determining whether the cannulae reach the ARH. Only the data of mice with correct cannula placement in the ARH were included in the analysis.

### 2.15 Measurement of LH

Blood samples were processed via an enzyme‐linked immunosorbent assay as reported previously.^25^ Monoclonal anti‐bovine LH beta subunit antiserum (RRID: AB_2665514) was purchased from University of California (CA, USA). Mouse LH standard (AFP‐5306A; dilution 1:25) and primary antibody (AFP240580Rb; dilution 1:40) were from NIDDK‐NHPP, USA. Following the incubation with the primary antibody (AFP240580Rb; dilution 1:40; NIDDK‐NHPP, USA), an enzyme‐linked secondary antibody (Donkey anti‐Rabbit, NA934VS ECL Peroxide Rb; VWR International (Lutterworth, UK)) binds the formed antibody–antigen complex. After incubation and washing steps to remove unbound material, a substrate solution containing o‐phenylenediamine (OPD) is added, which reacts with the peroxidase enzyme to generate a measurable color signal. The intensity of this OPD‐derived colorimetric signal is directly proportional to the concentration of the target analyte present in the original specimen. The intra‐assay and inter‐assay variations were 5.3% and 3.5%, respectively, and the assay sensitivity was 0.195 ng/mL.

### 2.16 Statistical analysis for in vivo experiments

Appropriate sample sizes were predetermined by way of power analyses that were performed using the SigmaStat software (Systat Software Inc., San Jose, CA, USA) and expected variances and effect sizes that were based on our preliminary studies. LH datasets were tested for normality and satisfied the assumptions for parametric analysis. A three‐way ANOVA was used to assess the main effects of group, dose, and light, and their interactions. The aCSF and 5 pmol groups were excluded from the three‐way ANOVA because of incomplete experimental conditions (absence of both light and no‐light conditions) and were analyzed separately using two‐way ANOVA. Statistical analyses were performed using GraphPad Prism (version 9; GraphPad Software, San Diego, CA, USA). ANOVA was used to compare mean and peak LH levels between experimental and control groups and to calculate the area under the curve. Data are presented as mean ± SEM, and statistical significance was defined as *p* < .05. The basal control period was defined as 0–30 min and the treatment period was defined as 30–60 min, where the infusion of *azo*‐senktide or aCSF vehicle occurred at 25 min and photostimulation began at 30 min and continued for 2 min. The blood sample at time point 30 min was collected before photostimulation began.

## 3 RESULTS

### 3.1 Synthesis and photophysical characterization of photoswitchable TacR3 agonist

**FIGURE 1:**
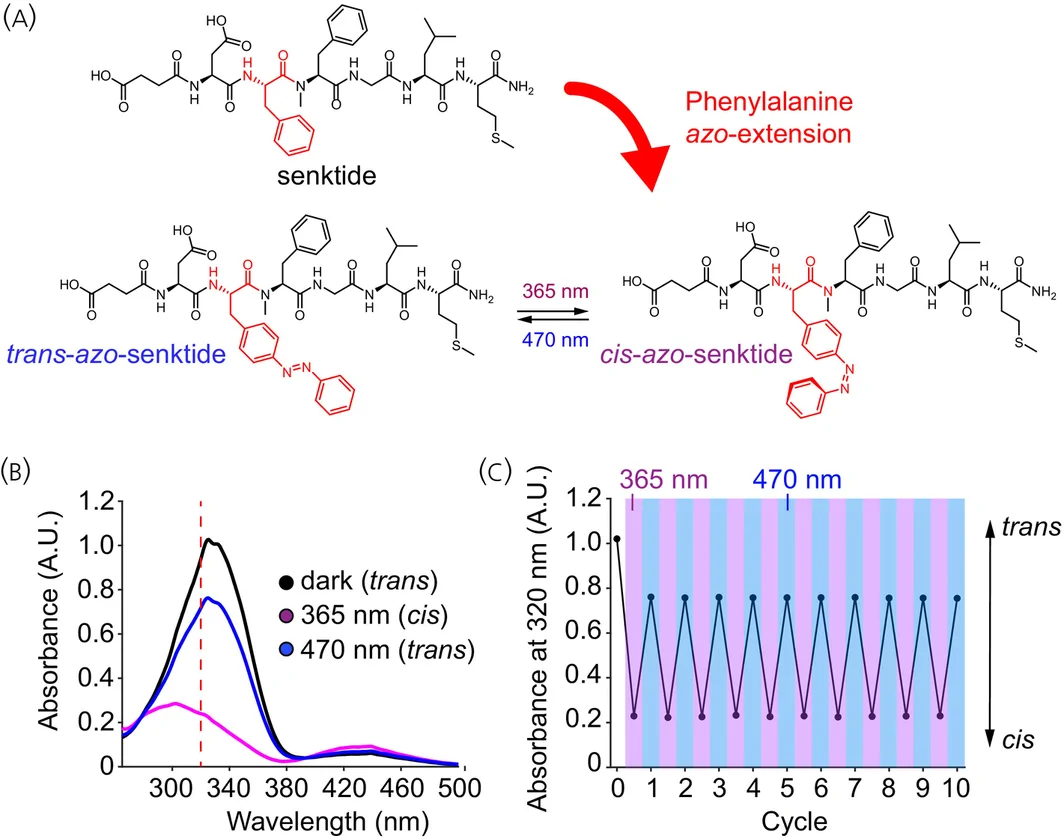
Design and photophysical characterization of a photoswitchable senktide. (A) Chemical structures of senktide (top) and *azo*‐senktide (bottom). *Azo*‐senktide can be isomerized between its *cis*‐ and *trans*‐states with UV‐A and blue light, respectively. (B) UV–vis spectra of *azo*‐senktide (40 μM in PBS buffer) in the dark‐adapted form (*trans*, black), after 3‐min 365‐nm irradiation (*cis*, purple) and after 3 min 470 nm irradiation (*trans*, blue). The red line indicates the absorbance value (at 320 nm) plotted in (C). (C) Absorbance value measured at 320 nm of a representative sample of *azo*‐senktide (40 μM in PBS buffer) after irradiated with 3‐min cycles of 365‐ and 470‐nm light. Photoswitching between *trans* and *cis* was reversible over many cycles. PBS, phosphate‐buffered saline.

Senktide, a potent and highly selective agonist for TacR3,^26^ is a small synthetic peptide (Asp‐Phe‐Pro‐[MePhe]‐Gly‐NH_2_) derived from the C‐terminal region of substance P and has been modified to enhance potency and selectivity^27^ (Figure 1A, top). Senktide serves as an ideal scaffold for developing a photoswitchable analog to agonize TacR3 due to a phenylalanine residue, where we envisioned expanding the aromatic side chain to include a phenyldiazine moiety. The resulting probe, *azo*‐senktide (Figure 1A, bottom), was synthesized via solid‐phase peptide synthesis, utilizing the azobenzene‐containing protected amino acid (Fmoc‐Phe(4‐N=NPh)—OH) to replace phenylalanine. The final peptide was purified by HPLC and confirmed to be pure by Liquid Chromatography‐Tandem Mass Spectrometry (LC–MS/MS) analysis (see Section 2 and Figures S1 and S2).

We carried out the photophysical characterization of *azo*‐senktide by UV–Vis spectroscopy. When in the dark and dissolved in aqueous buffer, *azo*‐senktide existed in the *trans*‐configuration and possessed the adsorption spectra of a standard azobenzene, with *λ*_max_ values at ~330 and 430 nm for the π➔π* and *n*➔π* electronic transitions, respectively (Figure 1B, black). Irradiation with 365‐nm LED light (~16 mW) caused the π➔π* transition to decrease in intensity and increased the *n*➔π* band (Figure 1B, pink), indicating that *azo*‐senktide had isomerized to *cis*. Subsequently, irradiation with 470 nm LED light (~16 mW) isomerized *azo*‐senktide back to *trans*, which increased the π➔π* and decreased n➔π* absorption bands (Figure 1B, blue and Figure S3B). Notably, photoswitching between *trans* and *cis* could be repeated over many cycles (Figure 1C). These results confirm that *azo*‐senktide is a photoswitchable peptide that can be isomerized between *cis* and *trans* with UV‐A and blue light in physiological buffer solution.

### 3.2 Azo‐senktide selectively activates TacR3 in HEK293T cells

**FIGURE 2:**
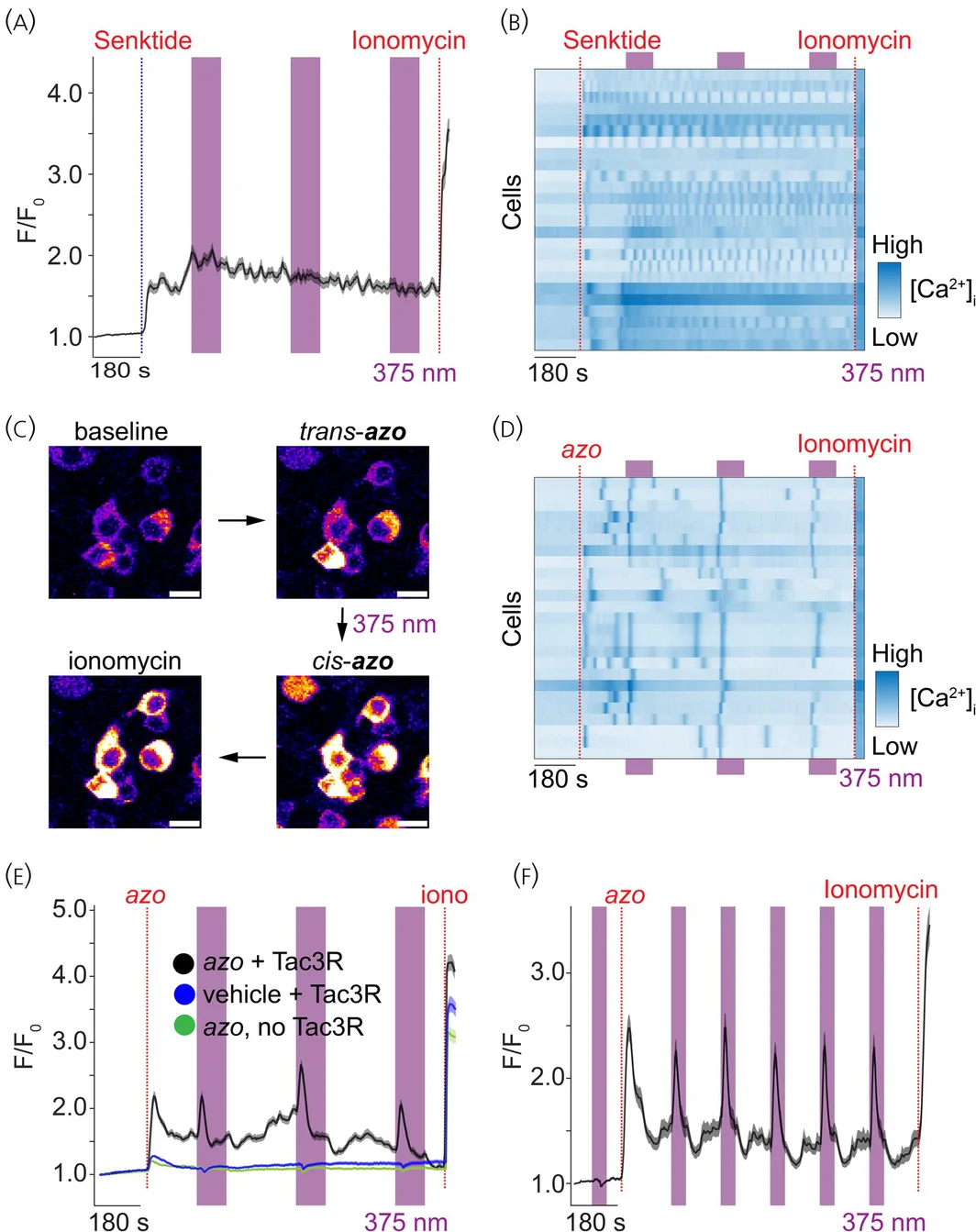
*Azo*‐senktide enables optical activation of TacR3‐mediated Ca^2+^ signaling in HEK293T cells. HEK293T cells were transfected with TacR3 and RGECO, then subjected to Ca^2+^ imaging by confocal microscopy. (A, B) Senktide (2 nM) was added to the cells subjected to three pulses of 375‐nm irradiation. A large Ca^2+^ influx and oscillations were observed on addition, but UV‐A irradiation had no effect. Shown as (A) averaged R‐GECO fluorescence (*N* = 137; *T* = 4); and (B) a heatmap from 25 individual cells. Ionomycin (10 μM) was added at the end to saturate Ca^2+^ levels. (C) Representative fluorescence images showing R‐GECO. Application of *trans*‐*azo*‐senktide (500 nM) caused a small Ca^2+^ increase, which was potentiated by 375‐nm irradiation (*t* = 204 s) and isomerization to *cis*. (D) Heatmap from individual representative cells treated with *trans‐azo*‐senktide and subjected to 3 pulses of 375 nm irradiation. (E) Average R‐GECO fluorescent trace for: (black) TacR3 overexpressing cells treated with *azo*‐senktide in (*N* = 211; *T* = 5); (green) *azo*‐senktide applied to HEK293T cells not transfected with TacR3 (*N* = 148; *T* = 3); and (blue) a vehicle addition (0.1% DMSO) applied to TacR3‐overexpressing cells (*N* = 148; *T* = 3). (F) *Azo*‐senktide allowed photostimulation over multiple 5 cycles with little desensitization (*N* = 50; *T* = 1). Shaded error bars = mean ± SEM. DMSO, dimethylsulfoxide.

To test the photoswitching efficacy of *azo*‐senktide at TacR3 in live cells, we employed a heterologous overexpression system where we monitored TacR3‐induced elevations in the intracellular Ca^2+^ concentration, [Ca^2+^]_*i*_. HEK293T cells do not express TacR3 endogenously,^28^ thus the cells were transfected with human‐TacR3 and the genetically‐encoded Ca^2+^‐sensor, R‐GECO.^29^ When imaged by live‐cell confocal fluorescence microscopy, application of senktide in the dark immediately caused a large increase in [Ca^2+^]_*i*_ and stimulated robust Ca^2+^ oscillations (Figure 2A,B). Ionomycin was added at the end of the experiment to saturate [Ca^2+^]_*i*_. Following, 375‐nm irradiation before or after senktide addition had no effect on Ca^2+^ levels, demonstrating that the HEK293T cells overexpressing TacR3, or senktide itself were not naturally sensitive to UV‐A irradiation.

We then applied *azo*‐senktide, which elicited a robust [Ca^2+^]_*i*_ increase on application, reflecting modest agonist activity of the dark‐adapted *trans*‐compound. However, irradiation with 375‐nm light caused an additional large elevation of [Ca^2+^]_*i*_ in the cells, indicating that the *cis*‐isomer is more potent and can further activate TacR3 (Figure 2C,D and Figure 2E, black). After the UV‐light was turned off, continuous RGECO imaging with 559‐nm light switched *azo*‐senktide back to *trans*, and then photoactivation of the probe could be repeated over multiple cycles. Up to five rounds of photostimulation could be achieved with only modest desensitization (Figure 2F). Vehicle addition had no effect on Ca^2+^ levels, even with photostimulation, confirming that light alone did not affect [Ca^2+^]_*i*_ (Figure 2E, blue and Figure S4A). In addition, *azo*‐senktide with photostimulation had no effect in HEK293T cells that were not transfected with the TacR3, confirming that the [Ca^2+^]_*i*_ response caused by *azo*‐senktide was selectively induced by the overexpressed TacR3 (Figure 2E, green and Figure S4B).

Overall, these results confirm that *azo*‐senktide is a photoswitchable TacR3 agonist that is activated by UV‐A light. Notably, azo‐senktide also produced a modest increase in intracellular Ca^2+^ prior to photostimulation, indicating that the dark‐adapted trans‐isomer retains a low level of agonist activity at TacR3. Photostimulation with 375‐nm light markedly enhanced this response, consistent with conversion to the more potent cis‐conformation. Therefore, optical activation enhances, rather than exclusively enables, TacR3 signaling, and suggests that limited baseline activity of the dark‐state compound cannot be excluded. While these results confirm that the *cis*‐isomer is indeed more potent at TacR3, they do not reflect the absolute potency of the pure trans or *cis*‐isomers because the competing RGECO and UV‐photoexcitation lasers will generate an unknown ratio of *cis*/*trans* isomers. Regardless, these results set the stage for application of *azo*‐senktide in more complex neuronal systems.

### 3.3 Photo‐switching of azo‐senktide excites Kiss1^ARH^ neurons

**FIGURE 3:**
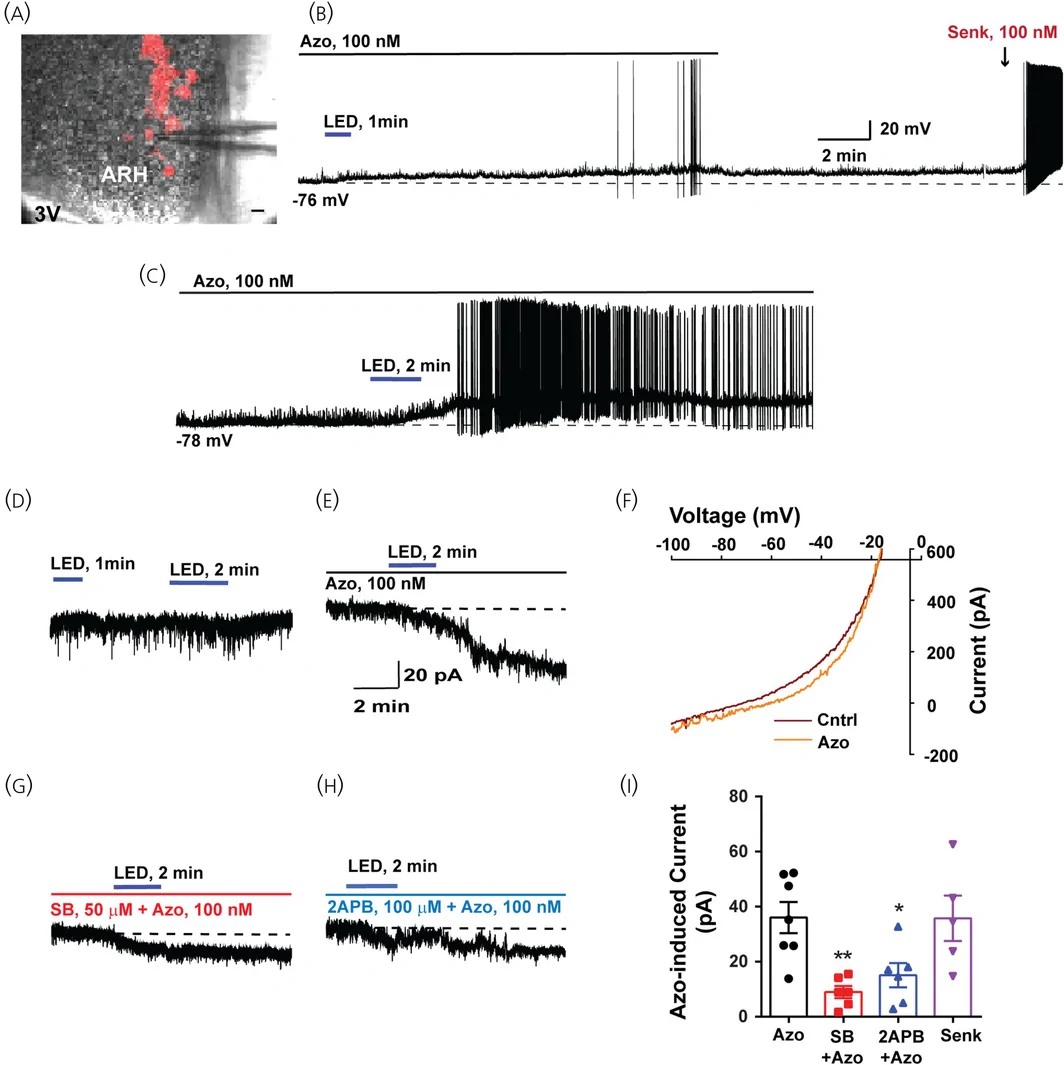
Photo‐switching of *azo*‐senktide excites Kiss1^ARH^ neurons. (A) Photomicrograph showing a recording pipette patched onto a Kiss1^ARH^‐mCherry cell. Scale bar, 20 μm. (B, C) Representative whole‐cell current‐clamp recordings showing membrane depolarization and action potential firing induced by photo‐switched *azo*‐senktide (100 nM) following exposure to a 375‐nm light pulse. After stable whole‐cell patch‐clamp recordings were established, slices were perfused and recirculated with *azo*‐senktide diluted in 40 mL of aCSF for at least 10 min to allow equilibration. Photostimulation was then applied. Neurons depolarized and exhibited increased firing following 1 min (B) or 2 min (C) of photostimulation. This response was mimicked by bath application of senktide (100 nM, B). In contrast, neurons did not respond to *azo*‐senktide in the absence of a 375‐nm light pulse. (D, E, G, H) Tepresentative voltage‐clamp recordings of the inward currents evoked in the absence (D) or presence (E) of *azo*‐senktide (100 nM), or in the presence of *azo*‐senktide together with the Tacr3 receptor antagonist SB222,200 (G) or the TRPC5 channel blocker 2‐APB (100 μM; H). (F) Current–voltage (I–V) relationship obtained before and during the peak response from the same cell shown in (E), indicating a reversal potential of approximately −20 mV for the nonselective cation current. (I) Bar graphs summarizing the effects of SB222,200 and 2‐APB on the *azo*‐senktide‐inward currents and showing the magnitude of the inward current evoked by senktide (100 nM). Comparisons between different treatments were analyzed using a one‐way ANOVA analysis (*F*_(3,20)_ = 6.9885, *p* = .0023) followed by Bonferroni's multiple comparisons test: *p < .05, **p < .01 versus control. Data expressed as mean ± SEM, with individual data points representing single neurons. 2‐APB, 2‐aminoethyl diphenylborinate; 3V, third ventricle; ANOVA, analysis of variance; ARH, hypothalamic arcuate nucleus; aCSF, artificial cerebrospinal fluid; LH, luteinizing hormone.

Next, to test whether azo‐senktide can be used to acutely control Kiss1 neuron excitability, we examined its effects on Kiss1^ARH^ neurons using whole‐cell electrophysiology. Acute ARH slices were prepared from adult female Kiss1^Cre^ mice that had received AAV1‐Ef1α‐DIO‐mCherry injection, and mCherry‐labeled Kiss1^ARH^ cells were targeted for current‐clamp and voltage‐clamp recordings (Figure 3A). Photoswitching of azo‐senktide (100 nM) reliably depolarized and increased firing of Kiss1^ARH^ neurons, and these effects were mimicked by senktide (100 nM) (Figure 3B). The magnitude of excitation depended on the duration of photostimulation, with 2 min of photostimulation producing a larger depolarization and firing response than shorter stimulation periods (Figure 3C). In voltage‐clamp recordings (*V*_hold_ = −60 mV), photostimulation alone for 1–2 min did not elicit any detectable inward current (Figure 3D). In contrast, in the presence of azo‐senktide, 2 min of photostimulation evoked a robust inward current (Figure 3E). The current–voltage (I/V) relationship of the photo‐evoked current closely matched that of the senktide‐induced current (Figure 3F), with a reversal potential near −20 mV as we reported before,^30^ consistent with activation of a non‐selective cation conductance. Pharmacological blockade further confirmed the signaling pathway involved. The inward currents induced by azo‐senktide photoswitching were attenuated by the TacR3 antagonist SB222,200 (Figure 3G,I) and by the TRPC channel blocker 2‐APB (Figure 3H,I). Together, these results demonstrate that photo‐switching of azo‐senktide selectively excites Kiss1^ARH^ neurons by activating TacR3 and produces responses consistent with engagement of the previously established TRPC5‐dependent signaling pathway.^4^, ^5^, ^6^

### 3.4 Photostimulation of azo‐senktide in the ARH drives pituitary release of LH in vivo

Next, we sought to apply *azo*‐senktide in vivo using chronically implanted optofluidic cannulas. We implanted cannulas into seven mice for delivery of azo‐senktide and 365‐nm light. Analysis of histological images acquired from coronal sectioning of the mouse brains showed that six out of seven females had successful cannula placement in the ARH. Data from the mouse with the misplaced cannula were excluded from the analysis but presented in Figure S5. A representative example of a brain section with the track of an optofluid cannula correctly positioned in the ARH is shown in Figure S6.

**FIGURE 4:**
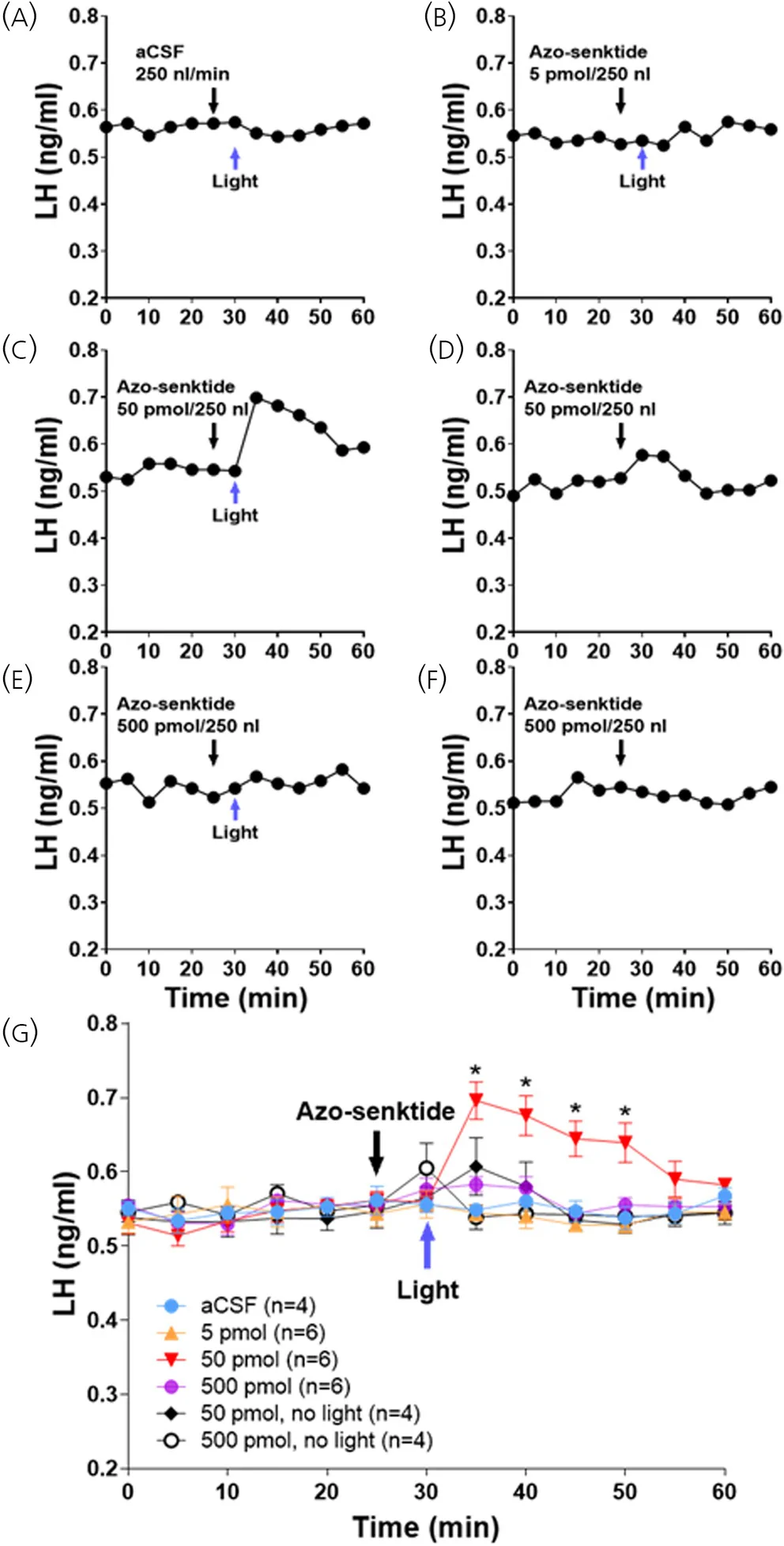
Dynamic changes in blood levels of LH after *azo*‐senktide administration with or without photostimulation in estrous female mice. (A) Representative example of intra‐arcuate nucleus (ARH) administration of artificial cerebrospinal fluid (aCSF) (250 nL) over 5 min followed by 2‐min photostimulation (continuous 365 nm at 7 mW LED); (B) 5 pmol *azo*‐senktide followed by 2 min photostimulation; (C) 50 pmol *azo*‐senktide followed by 2‐min photostimulation; (D) 50 pmol *azo*‐senktide without photostimulation; (E) 500 pmol *azo*‐senktide followed by 2‐min photostimulation; (F), 500 pmol *azo*‐senktide without photostimulation. (G) Summary of the dynamic changes in mean LH levels for all control and *azo*‐senktide treatment groups, showing a significant increase only in response to the 50 pmol *azo*‐senktide dose (red triangles) following photostimulation. Blood samples (4 μL) were collected every 5 min for 25 min prior to drug or aCSF infusion and for a further 35 min after treatment. All statistics conducted using a two‐way ANOVA. Data are represented as mean ± SEM. *p < .05, *n* = 4–6 per group. ANOVA, analysis of variance; ARH, hypothalamic arcuate nucleus; aCSF, artificial cerebrospinal fluid; LED, light‐emitting diode; LH, luteinizing hormone.

**FIGURE 5:**
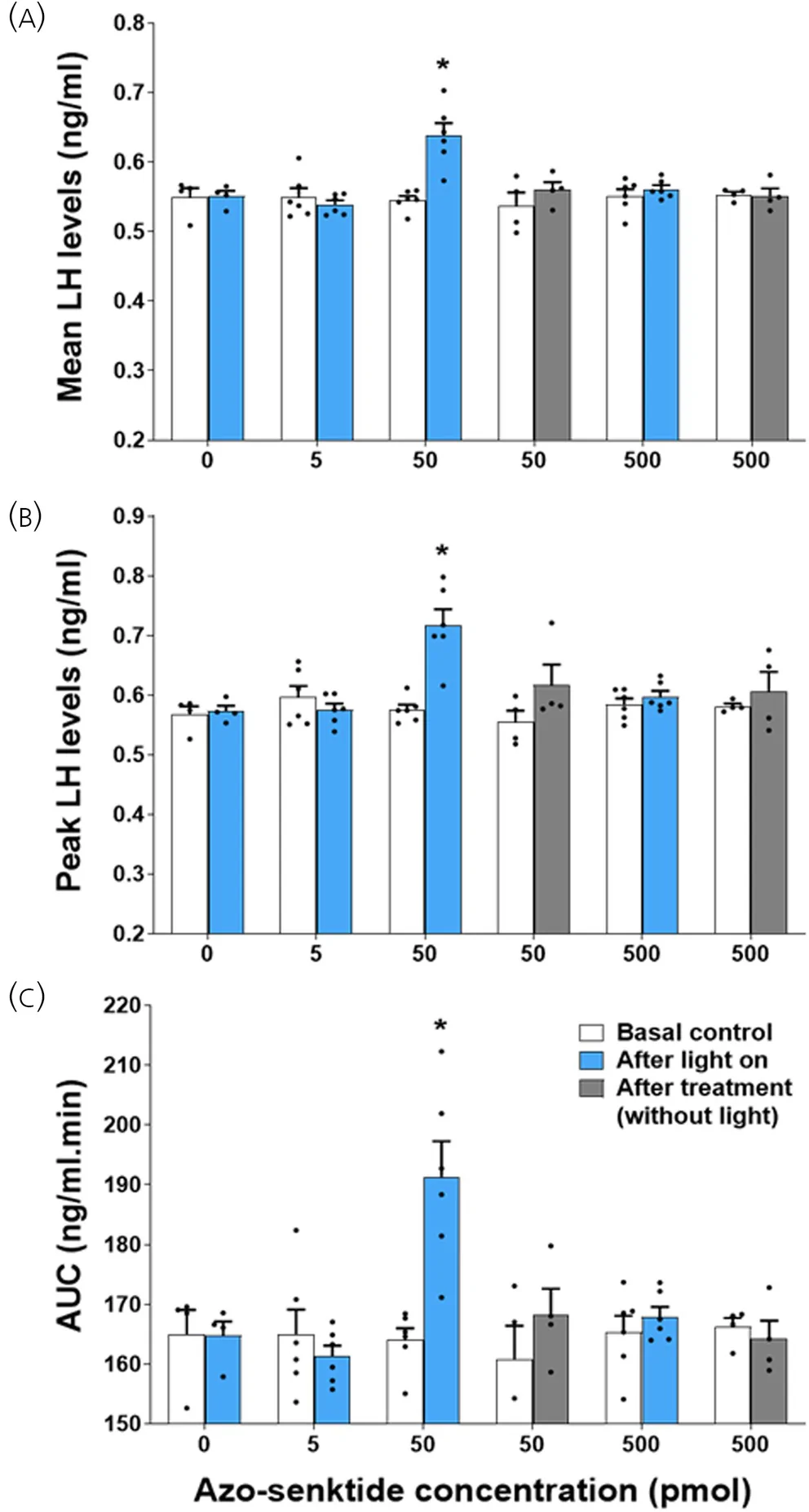
Effects of intra‐arcuate nucleus administration of *azo*‐senktide with or without photostimulation on LH secretory parameters in female estrous mice. These data represent the mean (A) and peak (B) LH levels and the AUC (C) for mice administered aCSF as control or 5 pmol *azo*‐senktide with photostimulation, and 50 pmol or 500 pmol *azo*‐senktide with and without photostimulation. The data show a statistically significant increase in serum LH levels after the 50 pmol dose with photostimulation compared to the other experimental and control groups. Results represent the mean ± SEM. *p < .01 versus its own control period and all other groups at the same treatment period. All statistics conducted using a two‐way ANOVA. *n* = 4–6 per group. aCSF, artificial cerebrospinal fluid; ANOVA, analysis of variance; AUC, area under the curve; LH, luteinizing hormone.

The dynamic changes in blood levels of LH in response to *azo*‐senktide administration with or without photostimulation are shown in Figure 4A–F. Although there was no effect of the low dose (5 pmol in 250 nL) of *azo*‐senktide on LH levels (Figure 4B,G), there was an immediate increase in LH release with the 50 pmol dose following photostimulation (Figure 4C,G). These post‐stimulus LH levels remained significantly elevated for 15 min compared to the other doses of the drug and the LH levels in the control groups (*p* < .01) (Figure 4G). However, there were minimal fluctuations with 50 pmol *azo*‐senktide in the absence of photostimulation in two out of four mice, and an example is shown in Figure 4D. Although these changes did not reach statistical significance, they are consistent with the modest TacR3 activation observed in HEK293T cells prior to photostimulation and suggest that the trans‐form of azo‐senktide may possess limited residual agonist activity in vivo. Photostimulation substantially amplified this response, indicating that conversion to the cis‐conformation enhances receptor activation and downstream LH release. Surprisingly, administration of 500 pmol *azo*‐senktide resulted in no significant increase with photostimulation (Figure 4E) possibly due to desensitization at this high dose. Also, 500 pmol *azo*‐senktide (without photostimulation) did not alter basal LH levels. The level of LH remained unchanged after administration of aCSF followed by light stimulation as a control (Figure 4A,G). A summary of the data representing mean and peak LH levels, and the area under the curve (AUC) in response to the three doses of *azo*‐senktide with and without photostimulation (2‐min continuous light at 365 nm, 7 mW) and aCSF as a vehicle control with photostimulation are shown in Figure 5A–C, respectively. The only significant change observed in mean and peak LH levels, and AUC was in response to intra‐arcuate nucleus administration of 50 pmol *azo*‐senktide with photostimulation (Figure 5A–C), all other doses did not alter LH levels.

## 4 DISCUSSION

The present study demonstrates that temporally controlled activation of TacR3 signaling is sufficient to drive LH pulse release in vivo and provides direct evidence that the physiological actions of neurokinin B (NKB) within the arcuate kisspeptin (Kiss1^ARH^) network depend critically on the pattern of receptor activation. Using a novel photoswitchable TacR3 agonist, azo‐senktide, we were able to reversibly control endogenous NKB signaling with light and thereby dissect the temporal requirements for activation of the GnRH pulse generator. Our findings reveal that optical activation of *azo*‐senktide selectively stimulates TacR3 signaling, excites Kiss1^ARH^ neurons in vitro through responses that are consistent with the previously described TacR3–TRPC5 signaling mechanism.^4^, ^5^, ^6^ and triggers a time‐locked single LH pulse in vivo. Together, these observations support the concept that NKB functions as an excitatory synchronizing signal within the KNDy network and suggest that previous conflicting observations regarding the effects of senktide in vivo^9^, ^10^, ^11^ can be explained by differences in the dynamics of TacR3 activation.

A major finding of this study is that photoactivation of *azo*‐senktide elicits cellular responses that closely resemble the established actions of endogenous NKB signaling in Kiss1^ARH^ neurons. Previous studies demonstrated that NKB activates TacR3 receptors expressed by Kiss1^ARH^ neurons and induces depolarization through activation of a TRPC5‐mediated nonselective cation conductance, thereby promoting neuronal synchronization and coordinated network activity.^4^, ^5^, ^6^ Consistent with these observations, photoisomerization of azo‐senktide evoked membrane depolarization, increased action potential firing, and generated inward currents with electrophysiological characteristics nearly identical to those induced by senktide. Furthermore, these responses were blocked by the TacR3 antagonist SB222,200 and the TRPC channel inhibitor 2‐APB, supporting the interpretation that the photoswitchable ligand acts through a mechanism consistent with the canonical TacR3–TRPC5 signaling pathway previously established in Kiss1^ARH^ neurons.^4^, ^5^, ^6^ Therefore, these findings validate azo‐senktide as a highly effective optical tool for probing the established TacR3 signaling pathway with precise temporal control.

The ability of optical activation of azo‐senktide to trigger a pulse of LH release in vivo provides important mechanistic insight into the operation of the GnRH pulse generator. Optogenetic stimulation of Kiss1^ARH^ neurons has previously been shown to evoke pulsatile LH secretion, supporting the hypothesis that these neurons form the core pulse‐generating network.^3^ However, optogenetic approaches bypass endogenous receptor signaling and directly depolarize neurons. In contrast, the present study demonstrates that selective activation of a native neuropeptide signaling pathway is sufficient to evoke hormone release. Importantly, only the intermediate dose of azo‐senktide produced an LH pulse response following photostimulation, whereas lower and higher doses were ineffective. The dose dependence observed in vivo suggests that successful recruitment of the KNDy network requires an optimal level of TacR3 activation capable of synchronizing neuronal activity without overwhelming the intrinsic mechanisms that regulate network oscillations.

The inverted‐U dose–response relationship observed in the present study should be interpreted within a broader physiological framework. Several mechanisms may contribute to the loss of efficacy at higher doses, including TacR3 receptor desensitization, nonlinear network dynamics within the KNDy neuronal population, and constraints at downstream levels of the reproductive axis. Increased hypothalamic excitation does not necessarily translate into a proportional increase in LH secretion. For example, excessive activation of KNDy neurons could potentially drive GnRH neurons beyond the optimal range for pulsatile output, while pituitary responsiveness may impose an additional limitation on LH release. Therefore, measurements of circulating LH, although representing the ultimate output of the reproductive neuroendocrine axis, may not fully reflect the complexity of upstream hypothalamic network activity. This interpretation is consistent with the possibility that enhanced activity within the pulse generator network can occur without a corresponding increase in LH secretion, highlighting the importance of considering multiple levels of regulation when interpreting neuroendocrine output.

These findings may help resolve a long‐standing paradox in the NKB field. Although NKB and senktide consistently excite Kiss1^ARH^ neurons in vitro and ex vivo, administration of senktide in vivo has been reported to suppress arcuate multiunit activity and inhibit LH pulsatility in several experimental settings.^10^, ^11^ The current results suggest that these apparently contradictory observations may reflect differences in the temporal characteristics of receptor activation rather than fundamentally different biological actions of NKB. Physiological NKB release within the KNDy network occurs in discrete bursts and is tightly coordinated with dynorphin‐mediated inhibitory feedback.^6^ In contrast, bolus administration of senktide likely produces prolonged and spatially widespread receptor activation that does not accurately mimic endogenous signaling dynamics. Sustained activation of TacR3 receptors may engage receptor desensitization mechanisms, recruit excessive dynorphin release, or disrupt the balance between excitatory and inhibitory signaling that is necessary for network oscillations. Consequently, prolonged receptor stimulation may suppress pulsatile activity even though the immediate cellular action of NKB is excitatory.

Current models of the KNDy pulse generator propose that NKB and dynorphin function as complementary positive‐ and negative‐feedback signals that coordinate synchronized activity within the Kiss1^ARH^ network.^1^, ^2^, ^31^ NKB‐mediated activation of TacR3 and TRPC5 channels promotes network excitation and synchronization,^4^, ^5^, ^6^ whereas dynorphin activates κ‐opioid receptors and GIRK channels to terminate bursts and establish inter‐pulse intervals.^4^ Computational and experimental studies have suggested that oscillatory output emerges from the dynamic interplay between these opposing signaling pathways.^6^, ^32^ The present findings strongly support this framework. Brief photoactivation of azo‐senktide likely reproduces the transient excitatory NKB signal that normally initiates synchronized network activity, resulting in an LH pulse. Conversely, excessive receptor activation at higher doses may shift the network toward a non‐oscillatory state through receptor desensitization and/or enhanced inhibitory feedback, thereby preventing effective pulse generation.

Beyond its implications for reproductive neuroendocrinology, this work highlights the power of photopharmacology for investigating neuropeptide signaling in complex neural circuits. Unlike conventional pharmacological approaches, photoswitchable ligands provide precise temporal control over receptor activation while preserving endogenous receptor expression and downstream signaling pathways. This feature is particularly valuable for studying oscillatory biological systems, where the timing and duration of receptor activation can be as important as the magnitude of stimulation.^33^ By enabling reversible optical control of TacR3 signaling in both brain slices and living animals, azo‐senktide provides a new experimental platform for dissecting the cellular and network mechanisms that govern pulsatile hormone secretion.

Several limitations of the present study should be considered. The optical stimulation protocol was designed to establish proof‐of‐principle that temporally gated TacR3 activation can drive LH pulse release, but it does not fully replicate the complex endogenous patterns of NKB release that occur during natural reproductive cycles. The latter would require examination of repeated photoactivation of azo‐senktide on LH pulse induction, an important future experimental paradigm. In addition, although the electrophysiological experiments identify TacR3–TRPC5 signaling as the primary mechanism underlying neuronal excitation, the downstream network interactions responsible for generating synchronized activity were not directly measured in vivo. Future studies combining photopharmacology with Ca^2+^ imaging using fiber photometry or single‐cell resolution GRIN‐lens miniendoscopy, or alternatively multi‐site electrophysiological recordings, will be valuable for determining how temporally controlled NKB signaling propagates through the KNDy network and ultimately drives GnRH secretion. Lastly, since the in vivo experiments were performed in wild‐type mice, the endocrine effects cannot be conclusively attributed to TacR3 activation specifically in Kiss1^ARH^ neurons. Contribution from other TacR3‐expressing cells within the arcuate nucleus, as well as local network or fiber‐of‐passage effects, are a distinct possibility and have to be considered. It should also be noted that the electrophysiological and endocrine experiments were performed in animals with different gonadal status. Specifically, slice recordings were obtained from ovariectomized females, whereas LH measurements were conducted in ovary‐intact estrous mice. Given the well‐established effects of ovarian steroids on KNDy neuron function and reproductive neuroendocrine output, these datasets were generated under distinct hormonal conditions and therefore should not be directly compared quantitatively. Nevertheless, both experimental approaches were designed to address complementary aspects of TacR3 signaling, namely its cellular actions on Kiss1^ARH^ neurons and its physiological consequences for LH release in vivo.

A major finding of this study is that photoactivation of azo‐senktide reproduces the established cellular actions of endogenous NKB signaling in Kiss1^ARH^ neurons. Previous studies demonstrated that NKB activates TacR3 receptors expressed by Kiss1^ARH^ neurons and induces depolarization through activation of a TRPC5‐mediated nonselective cation conductance, thereby promoting neuronal synchronization and coordinated network activity.^4^, ^5^, ^6^ Importantly, these studies were performed primarily in ex vivo preparations and therefore provide evidence for neuronal and network mechanisms rather than direct demonstration of endocrine oscillator control in vivo. In conclusion, the present study demonstrates that temporally restricted activation of TacR3 signaling is sufficient to excite Kiss1^ARH^ neurons in a manner consistent with the established TacR3–TRPC5 signaling model and trigger the release of an LH pulse in vivo. These findings may help reconcile previous discrepancies regarding the role of NKB in reproductive hormone regulation and support a model in which NKB serves as a physiological excitatory signal whose effectiveness depends on precise temporal control. More broadly, this work establishes photopharmacological manipulation of neuropeptide receptors as a powerful approach for investigating the mechanisms that govern neuroendocrine oscillators and other temporally organized neural circuits.

## AUTHOR CONTRIBUTIONS

**Yanyan Lin:** Investigation; methodology; writing – original draft; formal analysis. **Jian Qiu:** Conceptualization; investigation; writing – original draft; methodology; writing – review and editing; formal analysis; data curation; validation; visualization. **James A. Frank:** Conceptualization; investigation; funding acquisition; writing – original draft; methodology; validation; visualization; writing – review and editing; software; formal analysis; data curation; supervision; resources; project administration. **Victoria S. Halls:** Investigation; methodology; validation; formal analysis; data curation. **Antonio Muñoz:** Investigation; methodology; validation; formal analysis. **Janelle M. Tobias:** Investigation; methodology; validation; formal analysis. **Martin J. Kelly:** Conceptualization; investigation; funding acquisition; writing – original draft; methodology; validation; visualization; writing – review and editing; formal analysis; data curation; supervision; resources; project administration. **Kevin T. O'Byrne:** Conceptualization; investigation; funding acquisition; writing – original draft; writing – review and editing; supervision; resources; project administration. **Xiao Feng Li:** Conceptualization; investigation; funding acquisition; writing – original draft; methodology; validation; formal analysis; data curation; supervision. **Emily Bartram:** Investigation; writing – original draft.

## FUNDING INFORMATION

The authors acknowledge the financial contributions of the OHSU Medicinal Chemistry Core (Research Resource ID: SCR_019048). Compound synthesis was supported by the OHSU School of Medicine Faculty Innovation Fund Pilot Program (JAF). The electrophysiology ex vivo studies were supported by NIH Grant DK068098 (MJK). In vivo experimentation was funded by the BBSRC grant BB/005913/1 (KOB and XFL).

## CONFLICT OF INTEREST STATEMENT

The authors declare no conflicts of interest.

## Supporting information


**Figure S1.** HPLC of azo‐senktide: product eluting at 7.566 min and 10.490 min (*cis*/*trans*). HPLC: Agilent Technologies 1260 infinity II. Column: Agilent Pursuit XRs 100 Å C18, 4.6 × 250 mm, 5 μm. Flow rate: 1 mL/min. Gradient: 0–1 min 50% acetonitrile in water + 0.1% formic acid, 1–16 min 50–100% acetonitrile in water + 0.1% formic acid, 16–19 min 100% acetonitrile + 0.1% formic acid, 19–22 min 50% acetonitrile in water + 0.1% formic acid.

**Figure S2.** MS of azo‐Senktide: mass spectrum of HPLC peak eluting at 7.566 min (top) and 10.490 (bottom) min. ESI+ calculated m/z: [M + H]^+^ = 946.75, found: [M + H]^+^ = 946.08/946.10 (cis/trans). MS: Thermo Scientific LTQ Velos.

**Figure S3.** Kinetic analysis of azo‐senktide photoswitching by UV–Vis. Azo‐senktide photoswitching was analyzed by UV–Vis spectroscopy (40 μM in PBS buffer). (A) Dark adapted (trans) azo‐senktide was irradiated with 365‐nm light. The absorbance at 325 nm was plotted over time, demonstrating the decay kinetics and complete isomerization to the UV‐adapted photostationary state was observed after 3 min. (B) After irradiation from part A, azo‐senktide was irradiated with 470‐nm light. The absorbance at 325 nm increased, demonstrating complete photoswitching back to the trans‐enriched photostationary state in 3 min. *N* = 3 replicates for each. Normalized to *t* = 0 min. Shaded error bars = mean ± SEM.

**Figure S4.** Controls for Tac3R modulation of Ca^2+^ in HEK293T cells. HEK293T cells were transfected with or without TacR3, and RGECO, then subjected to Ca^2+^ imaging by confocal microscopy. (A) Application of a vehicle (0.1% DMSO) followed by 375 nm irradiation did not affect Ca^2+^ levels in HEK293T cells expressing Tac3R; shown as a heatmap for 25 representative cells. Ionomycin (10 μM) was added at the end to saturate Ca^2+^ levels. (B) In cells not transfected with Tac3R, *azo*‐senktide (500 μM) did not have an effect. Shown as a heatmap from representative cells. Shaded error bars = mean ± SEM.

**Figure S5.** Actual LH profiles from the single mouse with an off‐target optofluid cannula placement. Actual LH profiles from a single mouse with an off‐target optofluid cannula placement. (A–D) Dynamic changes in blood LH levels following intra‐arcuate nucleus administration of aCSF (250 nL) over 5 min (A), 5 pmol azo‐senktide (B), and 50 pmol azo‐senktide with (C) or without (D) photostimulation. Photostimulation (continuous 365 nm, 7 mW LED, 2 min) is indicated by blue arrows. Drug infusion is indicated by black arrows. Blood samples (4 μL) were collected every 5 min. These data are presented for illustrative purposes and were not included in the main statistical analysis.

**Figure S6.** Representative example of the optofluid cannula placement within the hypothalamic arcuate nucleus (ARH). Insert (top right corner) shown a schematic representation of the ARH and its special relationship to the other brain structures. The black arrow pointed to the tip position of an optofluid cannula in the ARH (areas within the dashed line). 3 V, third ventricle; LV, lateral ventricle.

## Data Availability

The data that support the findings of this study are available from the corresponding authors upon reasonable request.
