# Supplementary material for: Optical control of the hypothalamic arcuate nucleus kisspeptin neuronal network with a photoswitchable peptide that drives luteinizing hormone release in female mice
Source: J Neuroendocrinol. 2026 Sep 8;38(9):e70255. doi: 10.1111/jne.70255 (PMC13553209; doi:10.1111/jne.70255)
Supplement: Supplementary file 1 [file JNE-38-e70255-s001.pdf]

Figure S2

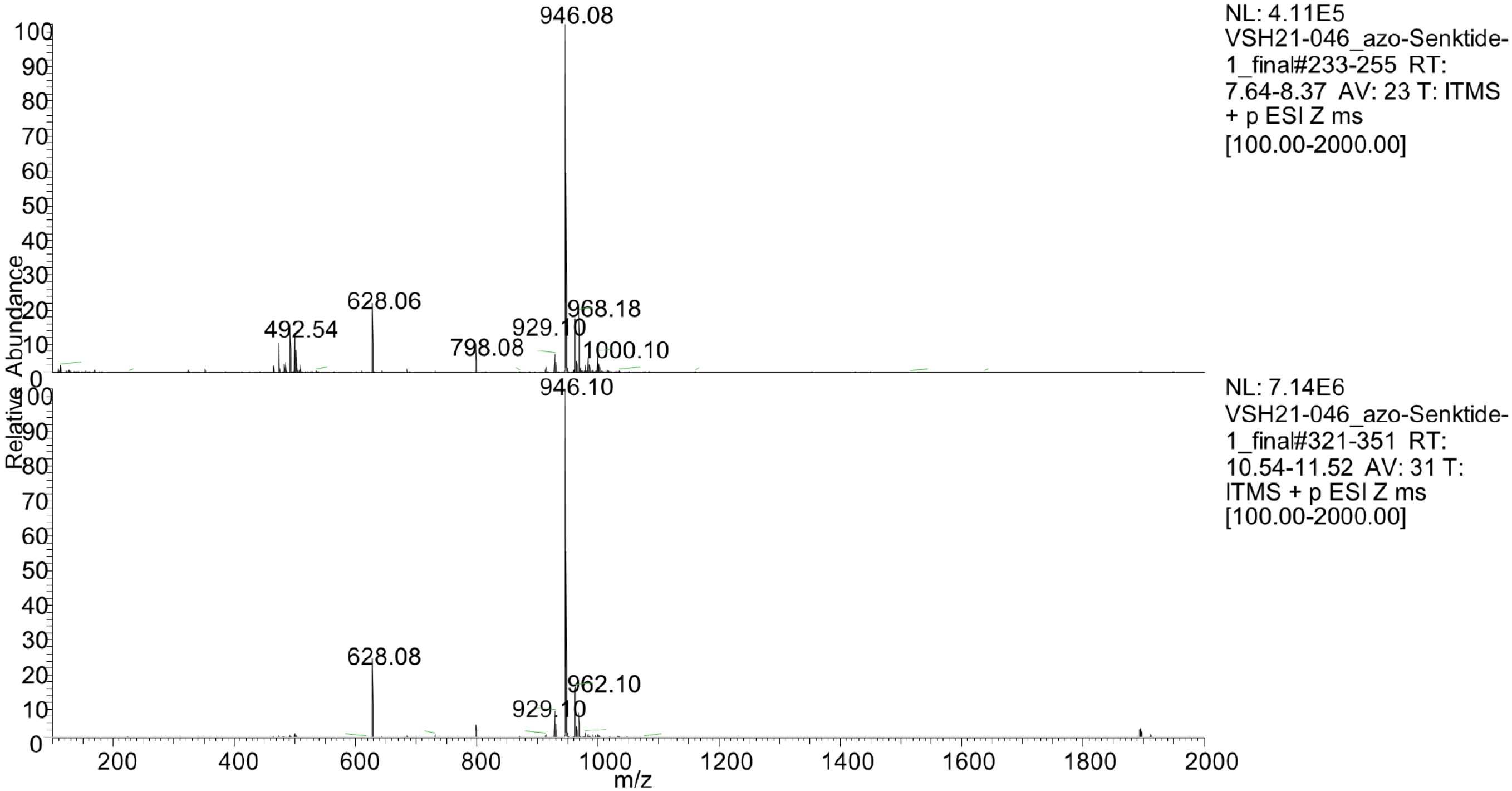
946.08
628.06
492.54
798.08
929.10
968.18
1000.10
NL: 4.11E5
VSH21-046_azo-Senktide-1_final#233-255 RT: 7.64-8.37 AV: 23 T: ITMS + p ESI Z ms [100.00-2000.00]
946.10
628.08
929.10
962.10
NL: 7.14E6
VSH21-046_azo-Senktide-1_final#321-351 RT: 10.54-11.52 AV: 31 T: ITMS + p ESI Z ms [100.00-2000.00]
Relative Abundance
m/z
0
10
20
30
40
50
60
70
80
90
100
200
400
600
800
1000
1200
1400
1600
1800
2000
