# Supplementary material for: Optical control of the hypothalamic arcuate nucleus kisspeptin neuronal network with a photoswitchable peptide that drives luteinizing hormone release in female mice
Source: J Neuroendocrinol. 2026 Sep 8;38(9):e70255. doi: 10.1111/jne.70255 (PMC13553209; doi:10.1111/jne.70255)
Supplement: Supplementary file 2 [file JNE-38-e70255-s002.pdf]

Figure S1

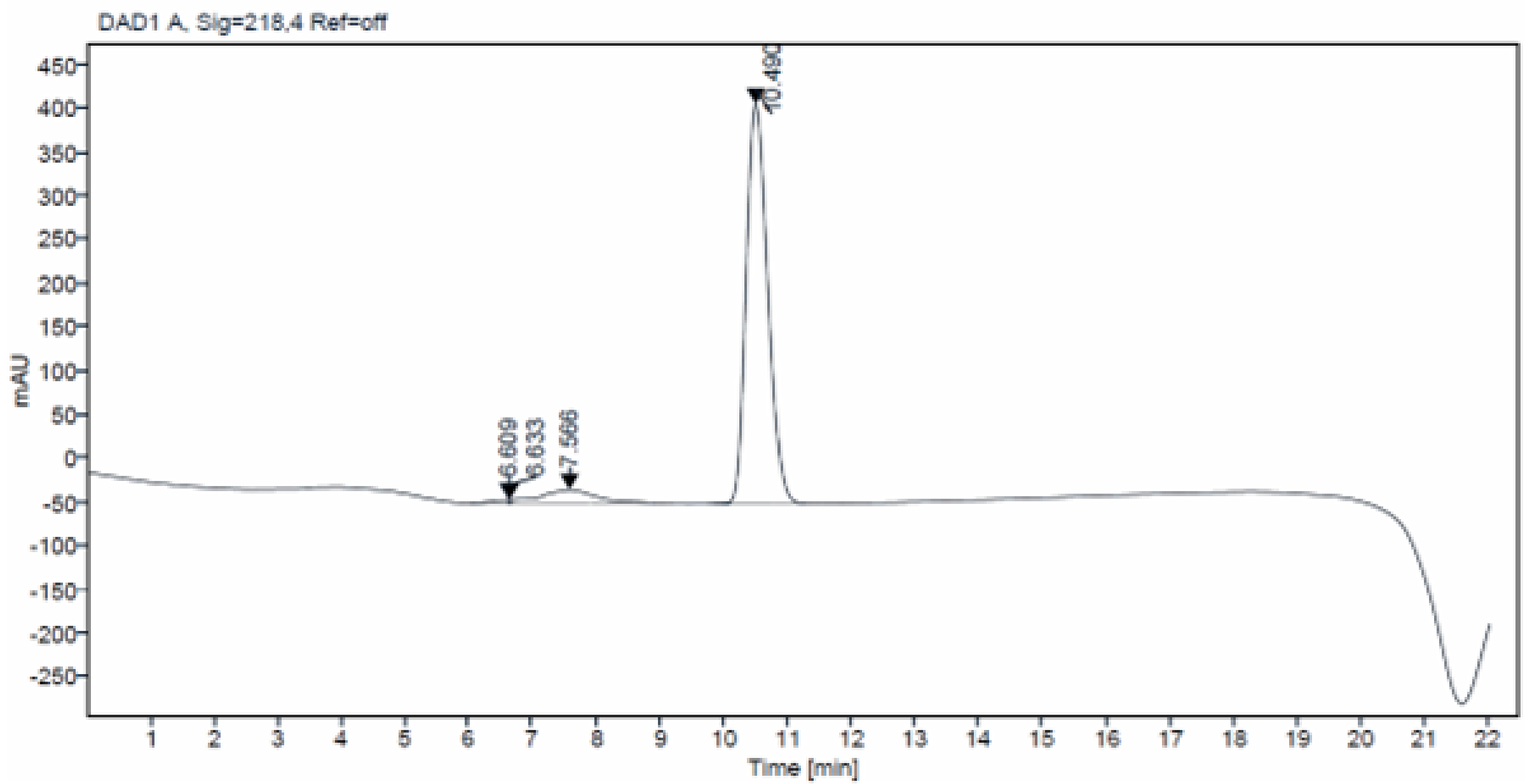

DAD1 A, Sig=218,4 Ref=off
mAU
450
400
350
300
250
200
150
100
50
0
-50
-100
-150
-200
-250
6.609
6.633
7.566
10.490
1
2
3
4
5
6
7
8
9
10
11
12
13
14
15
16
17
18
19
20
21
22
Time [min]
