# Supplementary material for: Optical control of the hypothalamic arcuate nucleus kisspeptin neuronal network with a photoswitchable peptide that drives luteinizing hormone release in female mice
Source: J Neuroendocrinol. 2026 Sep 8;38(9):e70255. doi: 10.1111/jne.70255 (PMC13553209; doi:10.1111/jne.70255)
Supplement: Supplementary file 3 [file JNE-38-e70255-s003.pdf]

Figure S4

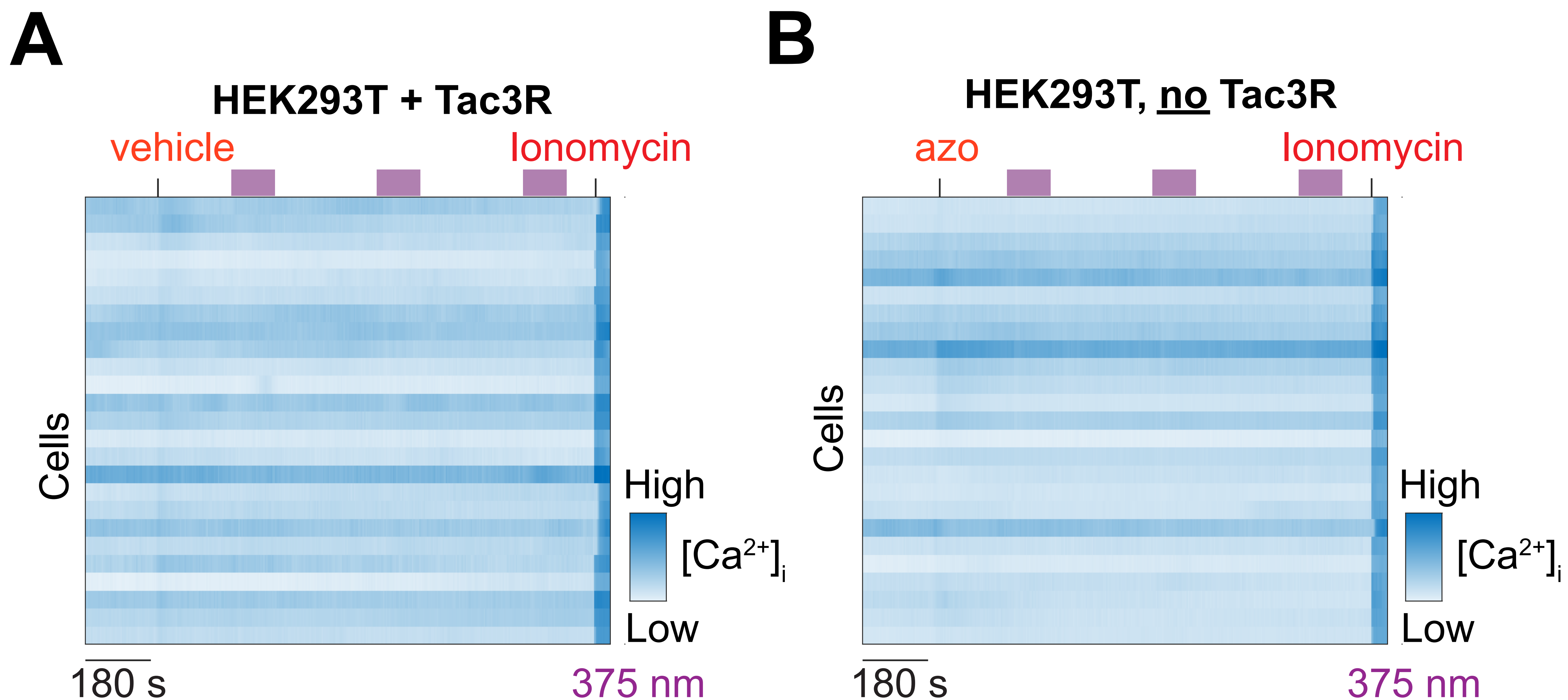
A
HEK293T + Tac3R
vehicle
Ionomycin
Cells
High
[Ca2+]i
Low
180 s
375 nm
B
HEK293T, no Tac3R
azo
Ionomycin
Cells
High
[Ca2+]i
Low
180 s
375 nm
