# Supplementary material for: Optical control of the hypothalamic arcuate nucleus kisspeptin neuronal network with a photoswitchable peptide that drives luteinizing hormone release in female mice
Source: J Neuroendocrinol. 2026 Sep 8;38(9):e70255. doi: 10.1111/jne.70255 (PMC13553209; doi:10.1111/jne.70255)
Supplement: Supplementary file 4 [file JNE-38-e70255-s005.pdf]

Figure S3

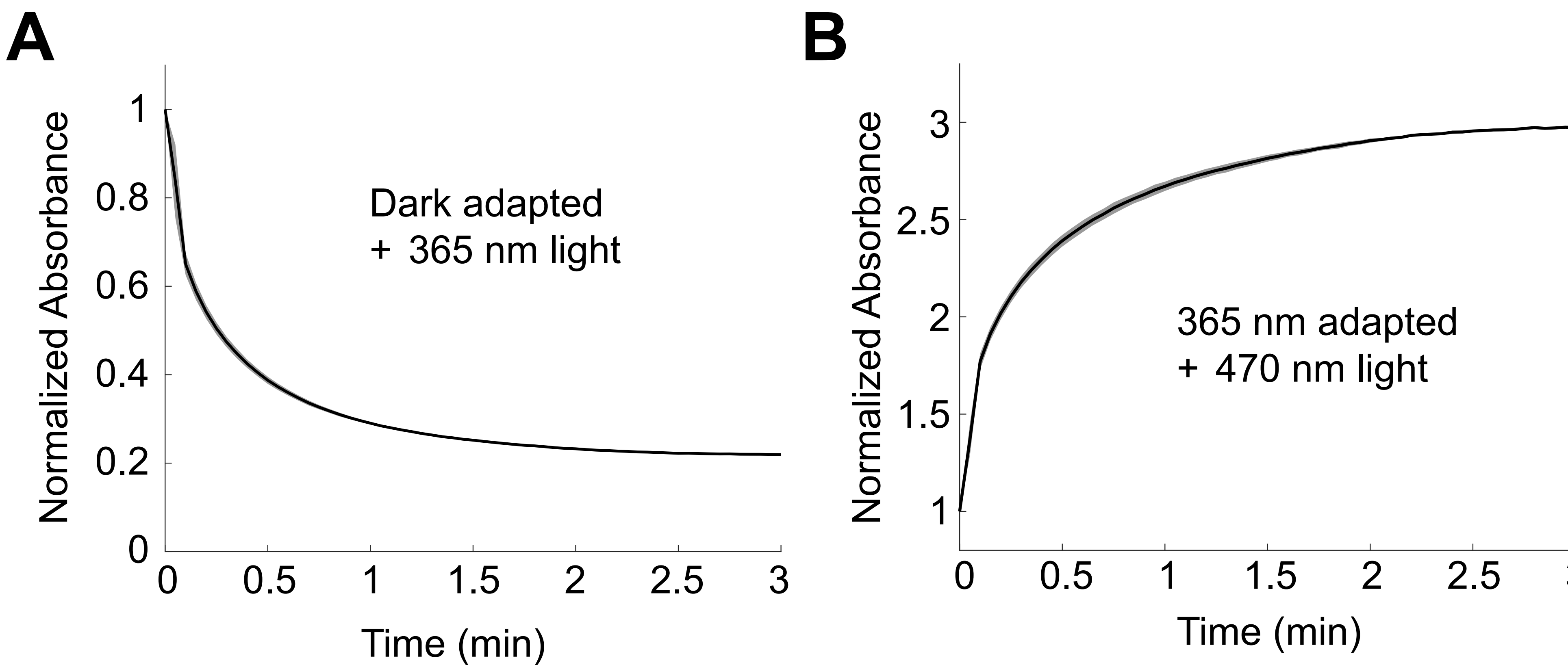
A
Normalized Absorbance
1
0.8
0.6
0.4
0.2
0
0
0.5
1
1.5
2
2.5
3
Time (min)
Dark adapted
+ 365 nm light
B
Normalized Absorbance
3
2.5
2
1.5
1
0
0.5
1
1.5
2
2.5
3
Time (min)
365 nm adapted
+ 470 nm light
